# Nonclassicality and Coherent Error Detection via Pseudo-Entropy

**DOI:** 10.3390/e27111165

**Published:** 2025-11-17

**Authors:** Assaf Katz, Shalom Bloch, Eliahu Cohen

**Affiliations:** Faculty of Engineering and the Institute of Nanotechnology and Advanced Materials, Bar-Ilan University, Ramat Gan 5290002, Israel

**Keywords:** quantum computing, quantum information, quantum error detection, pseudo-entropy, coherent errors

## Abstract

Pseudo-entropy is a complex-valued generalization of entanglement entropy defined on non-Hermitian transition operators and induced by post-selection. We present a simulation-based protocol for detecting nonclassicality and coherent errors in quantum circuits using this pseudo-entropy measure $\overset{ˇ}{S}$, focusing on its imaginary part $ℑ\overset{ˇ}{S}$ as a diagnostic tool. Our method enables resource-efficient classification of phase-coherent errors, such as those from miscalibrated CNOT gates, even under realistic noise conditions. By quantifying the transition between classical-like and quantum-like behavior through threshold analysis, we provide theoretical benchmarks for error classification that can inform hardware calibration strategies. Numerical simulations demonstrate that 55% of the parameter space remains classified as classical-like (below classification thresholds) at hardware-calibrated sensitivity levels, with statistical significance confirmed through rigorous sensitivity analysis. Robustness to noise and comparison with standard entropy-based methods are demonstrated in a simulation. While hardware validation remains necessary, this work bridges theoretical concepts of nonclassicality with practical quantum error classification frameworks, providing a foundation for experimental quantum computing applications.

## 1. Introduction

This paper presents a novel coherent error classification protocol for quantum systems, capable of diagnosing errors such as gate-phase errors arising from miscalibrated CNOT gates and general phase drifts. We lay the groundwork by introducing the pseudo-entropy formalism, its theoretical motivation from quantum field theory, and establishing the context within existing quantum error characterization research.

Recent advances in quantum noise characterization have produced scalable techniques such as measurement fidelity matrices (MFMs) and cumulant expansions [1], enabling comprehensive benchmarking of correlated and stochastic errors in large-scale quantum devices. Machine learning approaches also offer practical error mitigation strategies [2]. Additionally, assumption-free fidelity estimation frameworks [3] provide robust, model-independent measures of quantum device performance, addressing key challenges in validating quantum computational advantage. However, isolating specific coherent (unitary) error mechanisms remains challenging. Thus, our approach directly addresses this challenge by offering a resource-efficient, sensitive protocol specifically designed for classifying coherent errors, complementing and extending the capabilities of existing techniques.

### 1.1. Pseudo-Entropy Definition

Our approach uses pseudo-entropy, a generalization of entanglement entropy, computed from a reduced transition matrix $\rho_{\psi_{f}|\psi_{i}}^{\left(q_{0}\right)}$ on a qubit $q_{0}$. As established in recent studies on pseudo-entropy and non-Hermitian quantum mechanics [4,5,6], this matrix is defined as

(1) $$\rho_{\psi_{f}|\psi_{i}}^{\left(q_{0}\right)}={\mathrm{Tr}}_{q_{1}}\left(\frac{|\psi_{i}\rangle \langle \psi_{f}|}{\langle \psi_{f}|\psi_{i}\rangle }\right),$$

where ${\mathrm{Tr}}_{q_{1}}$ denotes the partial trace over $q_{1}$, and the denominator ensures normalization under post-selection. Unlike a standard Hermitian density matrix, the resulting $\rho_{\psi_{f}|\psi_{i}}^{\left(q_{0}\right)}$ is generally *non-Hermitian*.

The pseudo-entropy $\overset{ˇ}{S}$ is then defined as

(2) $$\overset{ˇ}{S}=-\mathrm{Tr}\left(\rho_{\psi_{f}|\psi_{i}}^{\left(q_{0}\right)}\log_{2}\rho_{\psi_{f}|\psi_{i}}^{\left(q_{0}\right)}\right),$$

where the logarithm is evaluated using the principal branch, accommodating the complex eigenvalues of $\rho_{\psi_{f}|\psi_{i}}^{\left(q_{0}\right)}$ [4,5].

Pseudo-entropy extends entanglement entropy to open, non-unitary systems, capturing coherent quantum features. Its imaginary part, $ℑ\overset{ˇ}{S}$, arises from the non-Hermitian nature of $\rho_{\psi_{f}|\psi_{i}}^{\left(q_{0}\right)}$ and serves as a sensitive indicator of coherent errors and nonclassical dynamics [5,7]. In ideal unitary evolution, $\rho_{\psi_{f}|\psi_{i}}^{\left(q_{0}\right)}$ remains Hermitian and $ℑ\overset{ˇ}{S}=0$. Coherent errors, such as systematic phase miscalibrations in CNOT gates, break Hermiticity, introducing complex eigenvalues whose phases contribute to non-zero $ℑ\overset{ˇ}{S}$. This makes $ℑ\overset{ˇ}{S}$ a direct witness of coherent, non-unitary behavior, consistent with interpretations from pseudo-Hermitian quantum mechanics [4,5].

A small-angle expansion of $\overset{ˇ}{S}$, discussed below, is useful for analyzing subtle coherent error regimes. The proposed protocol complements matrix-based and machine learning approaches by offering a gate-specific, resource-efficient diagnostic for coherent errors in quantum devices.

### 1.2. Theoretical Motivation for Coherent Error Detection

Pseudo-entropy, as a generalization of entanglement entropy, extends its theoretical utility beyond purely Hermitian systems to rigorously characterize nonclassical correlations and non-unitary dynamics. Its origins lie in quantum field theory (QFT) and non-Hermitian quantum mechanics, where it provides profound insights into phenomena such as entanglement in out-of-equilibrium systems, weak measurements, and the signatures of quantum chaos [5,6,7,8]. Unlike standard entanglement entropy, pseudo-entropy can be a complex-valued quantity, with its imaginary component specifically emerging from the non-Hermitian nature of generalized transition matrices that arise in scenarios involving post-selection or non-unitary evolution. This complex structure is not merely a mathematical artifact but a direct theoretical signature of coherent, non-Hermitian dynamics.

The theoretical motivation for employing pseudo-entropy in coherent error classification stems from this fundamental property: coherent errors, such as miscalibrated gate rotations or unintended phase accumulation, inherently introduce non-Hermitian elements into the effective evolution of a quantum system. In an ideal, perfectly unitary quantum computation, the effective evolution would be Hermitian, and the imaginary component of pseudo-entropy would theoretically vanish ($ℑ\overset{ˇ}{S}\approx 0$). Conversely, any deviation from this ideal, particularly coherent ones, will manifest as a non-zero $ℑ\overset{ˇ}{S}$. This provides a direct theoretical link between coherent errors and a quantifiable, interpretable metric.

Our protocol employs this pseudo-entropy-based classification framework to operationally enable error detection: by categorizing errors as classical-like or quantum-like based on a hardware-calibrated classification threshold, we consistently detect coherent phase errors once they exceed a critical magnitude, thus bridging theoretical classification with practical detection.

The underlying quantum circuit formalism is inspired by quantum field theoretical models such as [4], where the generalized transition operator is defined. Our experimental implementation yields a pseudo-density operator, ρ, whose value is determined by the control parameters $\left(\beta ,\delta \right)$ as defined in Section 2.1.

While pseudo-entropy is defined by Equation (2), we employ post-selection as a filter that selects an ensemble of states corresponding to specific measurement outcomes. This filtering process enables reconstruction of the transition matrix, where probabilities are computed from the resulting filtered ensemble; see Section 2.1 for details.

The theoretical framework posits that by carefully designing a quantum circuit, we can map specific types of coherent errors to the non-Hermitian properties of a reduced transition matrix. This allows for the theoretical definition of a classical-like region where $ℑ\overset{ˇ}{S}$ is below a certain threshold (indicating negligible coherent error) and a quantum-like region where a significant $ℑ\overset{ˇ}{S}$ indicates a detectable coherent deviation is indicated. This approach offers a theoretical basis for continuous, real-time monitoring of gate fidelity, enabling immediate detection of calibration drifts and proactive maintenance of quantum computer performance, moving beyond traditional post-facto characterization methods.

## 2. Materials and Methods

Building on the theoretical framework, we detail the experimental methodology and hardware calibration used in our pseudo-entropy-based coherent error detection protocol.

### 2.1. Application of Pseudo-Entropy for Error Detection

Unlike depolarizing or measurement-induced noise, which uniformly reduce state purity, coherent calibration errors modify phase relationships in measurable, directionally biased ways. As demonstrated through the pseudo-entropy (see Section 1), the discrete control parameters $\left(\beta ,\delta \right)$ in our circuit serve as effective surrogates for the corresponding continuous parameters, making pseudo-entropy diagnosis experimentally accessible.

In our framework, the parameters β and δ serve distinct but complementary roles in managing coherent errors. β represents a controlled rotation angle actively adjusted during the calibration process. Calibration involves systematically scanning β to minimize the magnitude of the imaginary component of pseudo-entropy, $\left|ℑ\overset{ˇ}{S}\right|$, thereby optimizing the intended interaction strength and reducing coherent phase errors. Conversely, δ models unknown systematic coherent phase deviations arising from hardware imperfections or drift. Benchmarking refers to the passive or ongoing monitoring of δ through measurements of $\left|ℑ\overset{ˇ}{S}\right|$ at fixed calibrated β values. This separation enables an efficient workflow: calibration actively optimizes system parameters by systematically varying β to minimize the measured error signature, specifically the imaginary part of pseudo-entropy (see Section 2.8 for details), while benchmarking provides continuous performance surveillance and error tracking (monitoring δ without parameter adjustment), signaling when recalibration is necessary.

Our protocol leverages the reduced matrix defined in Equation (1). When $\rho_{\psi_{f}|\psi_{i}}^{\left(q_{0}\right)}$ is diagonalizable, the trace-based expression admits a spectral decomposition:

(3) $$\overset{ˇ}{S}=-\sum_{k}\lambda_{k}\log_{2}\lambda_{k},$$

where $\lambda_{k}$ are the (possibly complex) eigenvalues of $\rho_{\psi_{f}|\psi_{i}}^{\left(q_{0}\right)}$, and the logarithm is taken using the principal branch for complex arguments. While this spectral decomposition facilitates theoretical interpretation and numerical computation, it may require careful numerical treatment. A non-zero imaginary part $ℑ\overset{ˇ}{S}$ reflects the presence of coherent, non-Hermitian dynamics and serves as a sensitive indicator of phase-calibration errors in quantum gates.

For small coherent errors, the imaginary part of pseudo-entropy admits a Taylor expansion:

(4) $$ℑ\overset{ˇ}{S}\left(\beta ,\delta \right)\approx A\beta^{2}+B\beta \delta +C\delta^{2}+D\beta +F\delta +G,$$

where the coefficients are defined as

(5) $$\begin{array}{cccccc} G & =ℑ\overset{ˇ}{S}\left(0,0\right), & D & ={\left.\frac{\partial ℑ\overset{ˇ}{S}}{\partial \beta }\right|}_{\left(0,0\right)}, & F & ={\left.\frac{\partial ℑ\overset{ˇ}{S}}{\partial \delta }\right|}_{\left(0,0\right)},\\ A & =\frac{1}{2}{\left.\frac{\partial^{2}ℑ\overset{ˇ}{S}}{\partial \beta^{2}}\right|}_{\left(0,0\right)}, & B & ={\left.\frac{\partial^{2}ℑ\overset{ˇ}{S}}{\partial \beta \partial \delta }\right|}_{\left(0,0\right)}, & C & =\frac{1}{2}{\left.\frac{\partial^{2}ℑ\overset{ˇ}{S}}{\partial \delta^{2}}\right|}_{\left(0,0\right)}. \end{array}$$

The notation $\left(0,0\right)$ refers to the origin in the two-parameter space $\left(\beta ,\delta \right)$, representing zero coherent rotation and interaction strength. This corresponds to the idealized regime in which the transition is perfectly unitary and error-free, and both the real and imaginary parts of pseudo-entropy are analytically shown to take their baseline values, specifically $ℑ\overset{ˇ}{S}\left(0,0\right)=0$ due to Hermiticity [4].

Our simulation analysis demonstrates that the coefficient *F* is a particularly strong indicator of coherent errors, providing a direct link between the physical error and the observed pseudo-entropy value.

### 2.2. Full Circuit and Measurement Strategy

Our protocol computes pseudo-entropy through a four-stage process:

1. State preparation initializes qubit $q_{0}$ in the |+〉 superposition state via Hadamard and qubit $q_{1}$ in |1〉 via an *X* gate, yielding the initial state $|\psi_{i}\rangle =|+1\rangle$.
2. Entanglement generation applies a CNOT gate with $q_{0}$ as control and $q_{1}$ as target to produce a Bell-like state. Coherent errors on the target qubit $q_{1}$ are modeled by the virtual rotation sequence $R_{Y}\left(\beta \right)\;R_{Z}\left(\beta +\delta \right)\;R_{X}\left(\delta \right)$, where β parametrizes the intended interaction (controlled rotation strength) and δ denotes a small systematic phase offset (miscalibration).
3. A final $R_{Z}\left(\frac{\pi }{2}\right)$ gate ensures numerical stability by maintaining sufficient overlap $\langle \psi_{f}|\psi_{i}\rangle$.
4. Tomographic measurement performs joint Pauli basis measurements over both qubits, enabling reconstruction of the reduced transition matrix $\rho_{\psi_{f}|\psi_{i}}^{\left(q_{0}\right)}$ via post-selection on measurement outcomes and partial trace over $q_{1}$.

**Figure 1:**
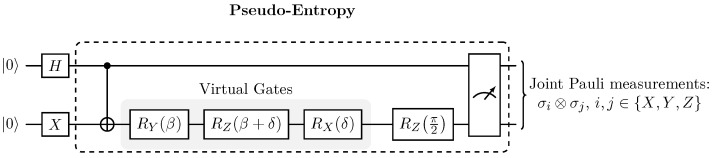
Quantum circuit implementing the pseudo-entropy measurement protocol. Initial state |+1〉 is prepared via Hadamard (H) on $q_{0}$ and bit-flip (X) on $q_{1}$. The CNOT gate creates entanglement, followed by parametrized error injection through virtual gates $R_{Y}\left(\beta \right)$, $R_{Z}\left(\beta +\delta \right)$, and $R_{X}\left(\delta \right)$ on $q_{1}$, where β represents controlled interaction strength and δ models systematic coherent offsets. A stabilizing $R_{Z}\left(\pi /2\right)$ rotation maintains numerical stability. Joint Pauli tomography enables reconstruction of the reduced transition matrix $\rho_{\psi_{f}|\psi_{i}}^{\left(q_{0}\right)}$ via post-selection and partial trace. Complete implementation is provided in pseudo_entropy.ipynb in GitHub [9].

This architecture isolates coherent CNOT errors while maintaining compatibility with NISQ hardware constraints. Figure 1 illustrates the complete measurement circuit implementing this protocol.

To compute the pseudo-entropy $\overset{ˇ}{S}$, we analyze the evolution from the initial state $|\psi_{i}\rangle$ to the final state $|\psi_{f}\rangle$, followed by joint tomography on both qubits. Taking the partial trace over qubit $q_{1}$ defines a conditional reduced state on qubit $q_{0}$, which isolates coherent effects caused by weak interactions and control imperfections.

Figure 1 shows the complete measurement circuit implementing this protocol, consisting of initial state preparation, a controlled entangling gate, a parameterized single-qubit operation modeling coherent errors, and joint tomographic measurements across all Pauli bases.

The quantum circuit (Figure 1) is designed to probe coherent CNOT errors by evolving an initial product state to an entangled Bell-like state, $|\psi_{i}\rangle =|+\rangle ⊗|1\rangle ,$ where $|+\rangle =\frac{|0\rangle +|1\rangle }{\sqrt{2}}$ is prepared via a Hadamard gate on $q_{0}$ and |1〉 via an *X* gate on $q_{1}$. The entanglement is performed with the CNOT gate,

(6) $$\mathrm{CNOT}|\psi_{i}\rangle =\frac{1}{\sqrt{2}}\left(|01\rangle +|10\rangle \right).$$

Coherent CNOT phase errors are modeled by a virtual gate sequence acting on $q_{1}$ (see Section 2.4):

(7) $$U_{q_{1}}\left(\beta ,\delta \right)=R_{Z}\left(\frac{\pi }{2}\right)R_{X}\left(\delta \right)R_{Z}\left(\beta +\delta \right)R_{Y}\left(\beta \right),$$

where β encodes the intended rotation and δ models systematic coherent offsets from miscalibration. The full pre-measurement state is

(8) $$|\psi^{\mathrm{pm}}\left(\beta ,\delta \right)\rangle =\left(I⊗U_{q_{1}}\left(\beta ,\delta \right)\right)\mathrm{CNOT}|\psi_{i}\rangle .$$

The final state $|\psi_{f}\rangle$ is determined experimentally from tomography following post-selection on measurement outcomes (see Section 2.6). Since $|\psi_{f}\rangle$ incorporates non-coherent noise that cannot be modeled analytically, we treat it as a measured quantity independent of the coherent error parameters $\left(\beta ,\delta \right)$ in our model. The coherent error parameters influence the transition amplitudes encoded in the tomographically reconstructed $\rho_{\psi_{f}|\psi_{i}}^{\left(q_{0}\right)}$ matrix, from which we extract δ through pseudo-entropy analysis.

### 2.3. Impact of Initial State

The sensitivity of pseudo-entropy $\overset{ˇ}{S}$ to coherent CNOT phase errors appears to depend critically on the chosen initial state and gate configuration. Our protocol initializes the system in |+1〉=|+〉⊗|1〉. This maximally entangled input appears to enhance the sensitivity of $\overset{ˇ}{S}$ to coherent distortions introduced after the entangling step.

In our simulations, alternative initial states (e.g., |00〉 and |++〉) produced substantially smaller pseudo-entropy signals. This motivated the use of |+1〉, whose entanglement structure appears to offer improved sensitivity for the circuits and noise models considered. Preparing |+1〉 uses only standard initialization gates, adds no extra circuit overhead, and is straightforward to implement on common hardware. We note, however, that optimal state choice may depend on circuit specifics and device noise, so other initial states could be preferable in different experimental settings.

Thus, the gate configuration and initial state together create a diagnosable setting in which $\overset{ˇ}{S}$ acts as a sensitive indicator of CNOT phase error strength and symmetry. While δ models systematic coherent offsets that are typically not directly controllable, this interpretability is key to validating both the symbolic derivations and the classification threshold used in simulation.

### 2.4. Error Modeling and Alternative Parameterizations

Our framework accommodates a comprehensive approach to error modeling by considering parameterized rotations about multiple axes of the Bloch sphere, including $R_{Z}$, $R_{X}$, and $R_{Y}$ gates. This multidimensional parametrization enables capturing a broad spectrum of coherent errors beyond simple phase errors around the *Z*-axis.

The choice of the virtual gate sequence $U_{q_{1}}\left(\beta ,\delta \right)=R_{Z}\left(\pi /2\right)R_{X}\left(\delta \right)R_{Z}\left(\beta +\delta \right)R_{Y}\left(\beta \right)$ is motivated by the decomposition of two-qubit gate errors into sequences of single-qubit coherent rotations. Specifically, CNOT calibration errors commonly manifest as unwanted single-qubit phase accumulations and over-rotations on the target qubit [10]. Equivalently, the core virtual error model

(9) $$U_{\mathrm{CNOT}\text{-}\mathrm{err}}\left(\beta ,\delta \right)=R_{X}\left(\delta \right)\;R_{Z}\;\left(\beta +\delta \right)\;R_{Y}\left(\beta \right),$$

applied to $q_{1}$, symbolically captures coherent over-/under-rotations that occur within hardware CNOT implementations due to miscalibration. Placing this effective error on the CNOT target allows even subtle modifications to the entangled state’s coherence structure to be reflected in the reduced transition matrix of $q_{0}$. In our implementation, we prepend an $R_{Z}\;\left(\pi /2\right)$ for numerical stability so that $U_{q_{1}}\left(\beta ,\delta \right)=R_{Z}\;\left(\pi /2\right)U_{\mathrm{CNOT}\text{-}\mathrm{err}}\left(\beta ,\delta \right)$.

Here, β represents the tunable interaction strength used during calibration sweeps, and δ models systematic coherent offsets arising from hardware imperfections such as flux noise or microwave amplitude drift. This error model affords both analytical tractability and physical relevance, allowing direct correlation of measured pseudo-entropy variations to underlying calibration parameters.

In addition to coherent errors, stochastic errors and state preparation and measurement (SPAM) errors are acknowledged, following prevalent frameworks in quantum error characterization. Techniques such as randomized compiling can convert coherent error components into effectively stochastic noise, enhancing diagnostic robustness. Stochastic errors, such as decoherence and measurement noise, are incorporated separately through realistic IBM hardware noise models in our simulations.

While the current implementation focuses on combining two-qubit CNOT gates with an additional calibrated $R_{Z}\left(\pi /2\right)$ rotation for phase-sensitive error classification and numerical stability, this setting serves as a foundational building block. Future extensions envision adapting and calibrating sequences involving various single-qubit rotations parameterized flexibly to capture and mitigate a richer set of operational errors empirically.

This layered parametrization approach is crucial for realizing scalable quantum characterization and correction protocols suitable for the heterogeneous and dynamic noise environments of current and forthcoming quantum computing hardware.

### 2.5. Role of the Final $R_{Z}\left(\pi /2\right)$ Gate

The final $R_{Z}\left(\pi /2\right)$ gate enhances numerical stability and interpretability by maintaining a sufficiently large overlap $\langle \psi_{f}|\psi_{i}\rangle$, preventing instabilities associated with division by near-zero values during post-selection while preserving phase error sensitivity. This ensures reliable classification of subtle coherent phase errors.

### 2.6. Tomography Procedure

Our protocol reconstructs the reduced transition matrix $\rho_{\psi_{f}|\psi_{i}}^{\left(q_{0}\right)}$ using full two-qubit tomography with post-selection, requiring only the two principal qubits; no ancillas are introduced. This approach relies on joint measurements and classical postprocessing, minimizing hardware overhead.

Our protocol uses a set of nine measurement settings, corresponding to all possible combinations of measuring each qubit in the *X*, *Y*, or *Z* basis (3×3=9). This excludes direct measurement configurations involving identity (*I*) operators. For each such measurement, summation over the full probability distribution allows reconstruction of elements not directly accessible through *I*. For each setting, all four post-selection $\left(|00\rangle ,|01\rangle ,|10\rangle ,|11\rangle \right)$ outcomes are considered. This strategy is theoretically complete and efficient for reconstructing the reduced two-qubit state, minimizing measurement overhead in both simulation and experiment.

In simulation, we perform the nine Pauli measurements, repeating each setting multiple times to ensure statistical confidence for each possbile outcome $\left(|00\rangle ,|01\rangle ,|10\rangle ,|11\rangle \right)$. Post-selection filters outcomes into conditioned subensembles defined by the transition operator $\rho_{\psi_{f}|\psi_{i}}^{\left(q_{0}\right)}$ (Equation (1)). Each post-selected state $|\psi_{f}\rangle$ is fixed per measurement outcome and independent of the unitary parameters β and δ, while $\rho_{\psi_{f}|\psi_{i}}^{\left(q_{0}\right)}$ depends on these parameters through transition amplitudes. The effective measurement overhead scales with post-selection success rate, noise, and desired precision, often requiring more than the nominal 36 settings.

Spectral decomposition of the reconstructed non-Hermitian matrix yields the eigenvalues used in pseudo-entropy calculation. The imaginary component, extracted per post-selected ensemble, directly signals coherent deviations [4]. This tomographic method enables accurate reconstruction of $\rho_{\psi_{f}|\psi_{i}}^{\left(q_{0}\right)}$ while accounting for noise and leveraging the parametric structure of the transition amplitudes.

### 2.7. Classification and Circuit Segmentation

To isolate coherent error signatures within the quantum circuit, we employ a custom segmentation protocol dividing the circuit into operational segments for pseudo-entropy analysis. Applying empirically calibrated thresholds, for example, 0.63% and 1.00% on the imaginary part $ℑ\overset{ˇ}{S}$, to these segments enables classification of behavior as classical-like or quantum-like. This segmentation is essential for detailed error mapping, and classical-like rates alongside local error indicators are quantified as described in Section 2.10.

### 2.8. Hardware Calibration and Classification Criteria

Performance validation utilizes IBM quantum circuit simulations via Qiskit’s FakeBackend with realistic noise models including readout errors, gate errors, and thermal relaxation. The classification of system behavior is conducted based on the pseudo-entropy metrics using the criterion:

(10) $$ℜ\overset{ˇ}{S}>0\;\mathrm{and}\;\left|ℑ\overset{ˇ}{S}\right|<\epsilon ,$$

where $ℜ\overset{ˇ}{S}$ captures classical contributions and $ℑ\overset{ˇ}{S}$ isolates coherent effects. The threshold ϵ is chosen consistent with measured readout error rates from IBM hardware [10], enabling discrimination between classical-like and quantum-coherent behavior.

### 2.9. Threshold Calibration and Justification

Thresholds for classifying classical-like versus quantum-like behavior are calibrated using IBM backend readout error rates. We adopt 0.63% (Washington backend) as a conservative lower bound and 1.00% (Algiers backend) as a typical operational benchmark, reflecting realistic NISQ hardware performance. These values are summarized in Table 1.

This calibration ensures a balance between sensitivity and specificity, avoiding excessive false negatives from overly lenient thresholds. At these calibrated levels, the classical-like rate stabilizes around 55%, indicating robustness to noise and numerical artifacts. Our simulation framework supports this calibration through controlled error injection, reproducible results, and systematic validation across the $\left(\beta ,\delta \right)$ parameter space. Our simulation framework supports this calibration through controlled error injection, reproducible results, and systematic validation across the $\left(\beta ,\delta \right)$ parameter space.

This statistical framing enhances interpretability and provides actionable confidence in the reported classification accuracy beyond simple heuristics like the area under the curve. At the 0.63% and 1.00% thresholds, classical-like rates consistently exceed 50%, confirming sensitivity to coherent errors rather than random noise. Each segment is sampled with approximately 400 points to ensure statistical reliability, and false positive rates are characterized within this framework.

Future work may explore adaptive thresholding strategies that respond dynamically to evolving hardware noise profiles, further improving detection fidelity.

### 2.10. Classical-like Rate Definition and Significance

Throughout this work, the term *classical-like rate* refers to the fraction of parameter points classified as classical-like (below the error classification threshold), not the fraction where errors are successfully detected.

Physical interpretation:

- *High classical-like rate (∼90%)*: Most errors in the sampled range are too small to detect (mostly classical-like, below threshold).
- *Low classical-like rate (∼10%)*: Most errors in the sampled range are large enough to detect (mostly quantum-like, above threshold).
- *Moderate classical-like rate (∼55%)*: Transition regime where roughly half the error magnitudes are detectable.

Only δ=0 represents a truly error-free CNOT gate. For any δ≠0, a coherent error exists. The classical-like rate quantifies what fraction of these errors remain below the protocol’s sensitivity threshold at a given error magnitude distribution. Small $\left|\delta \right|\to$ high classical-like rate (errors too small to see); large $\left|\delta \right|\to$ low classical-like rate (errors easily classified as quantum-like).

The ∼55% rate reported throughout indicates the protocol operates in a balanced sensitivity regime where it can distinguish significant errors while avoiding excessive false positives from noise.

To ensure statistical rigor, classical-like rates include Clopper-Pearson exact binomial confidence intervals [11] (CI), offering conservative and reliable coverage, even for small or skewed samples.

### 2.11. Simulation Parameters

Systematic parameter sweeps were performed for β ranging from −π to π with a step size of $1.25\times 10^{-3}\pi$ (1601 points), and for δ covering $-\frac{\pi }{128}$ to $\frac{\pi }{128}$ with a step size of $3.906\times 10^{-5}\pi$ (401 points). For further implementation details and full calibration data, refer to the computational notebook (pseudoentropy.ipynb) in GitHub repo [9].

### 2.12. Practical Implementation Parameters

Table 2 summarizes the key experimental parameters required for hardware deployment of this protocol. These specifications are informed by current IBM quantum hardware capabilities and represent realistic operational constraints for near-term devices.

These parameters reflect the minimal resource requirements for implementing the pseudo-entropy diagnostic on current superconducting quantum processors. The circuit depth and qubit count represent fundamental protocol constraints, while threshold values are calibrated to match realistic hardware noise profiles.

## 3. Results

Results confirm that phase error δ was sampled over a narrow range, reflecting our focus on the challenging regime of small, non-trivial coherent errors, where sensitive diagnostics like $ℑ\overset{ˇ}{S}$ provide the most value, as large δ values result in easily detectable, highly depolarized CNOT gates. All classical-like rates and classification results reported below are calculated using the segment analysis protocol described in Section 2.7.

### 3.1. Analysis of Quantum-Classical Regions and Performance Metrics

Classification performance is summarized in Table 3, which presents the classification accuracy (classical-like rates) for horizontal segments near δ=0 using two hardware-calibrated thresholds. These thresholds demonstrate robust classification capability, as the resulting rates significantly exceed random classification (50%).

### 3.2. Small-Angle Expansion of Pseudo-Entropy

To validate our framework, we compared the numerically obtained values of pseudo-entropy with analytical expressions. The numerical and analytical results show excellent agreement across the parameter space.

We analyze the sensitivity of pseudo-entropy with respect to small coherent errors by expanding the quantum circuit unitary $U_{q_{1}}\left(\beta ,\delta \right)$ to second order in the interaction strength β and coherent error parameter δ. The circuit applies

(11) $$U_{q_{1}}\left(\beta ,\delta \right)=R_{Z}\left(\frac{\pi }{2}\right)R_{X}\left(\delta \right)R_{Z}\left(\beta +\delta \right)R_{Y}\left(\beta \right)$$

to qubit 1, producing a final state

(12) $$|\psi_{f}\rangle =\left(I⊗U_{q_{1}}\left(\beta ,\delta \right)\right)\mathrm{CNOT}|\psi_{i}\rangle ,$$

where the initial state is $|\psi_{i}\rangle =\frac{1}{\sqrt{2}}\left(|01\rangle +|11\rangle \right)$.

The rotation matrices $R_{X}$, $R_{Y}$, and $R_{Z}$ are expanded using their Taylor series up to second order in angle:

(13) $$R_{X}\left(\theta \right)\approx \left(\begin{array}{cc} 1-\frac{\theta^{2}}{8} & -i\frac{\theta }{2}\\ -i\frac{\theta }{2} & 1-\frac{\theta^{2}}{8} \end{array}\right),$$

with analogous expansions for $R_{Y}\left(\theta \right)$ and $R_{Z}\left(\theta \right)$.

The combined unitary expands as

(14) $$U_{q_{1}}\left(\beta ,\delta \right)\approx U_{0}+\beta U_{1}+\delta U_{2}+\beta^{2}U_{11}+\beta \delta U_{12}+\delta^{2}U_{22}+O\left(\beta^{3},\delta^{3}\right).$$

Correspondingly, the final state and overlap expand perturbatively, enabling calculation of the generalized transition matrix and its partial trace on qubit 0 to obtain a reduced matrix ${\hat{\rho }}_{q_{0}}$, whose eigenvalues $\lambda_{k}$ expand as

(15) $$\lambda_{k}=\lambda_{k}^{\left(0\right)}+\beta \lambda_{k}^{\left(1\right)}+\delta \lambda_{k}^{\left(2\right)}+\beta^{2}\lambda_{k}^{\left(11\right)}+\beta \delta \lambda_{k}^{\left(12\right)}+\delta^{2}\lambda_{k}^{\left(22\right)}+O\left(\beta^{3},\delta^{3}\right).$$

The pseudo-entropy is then approximated by a second-order polynomial:

(16) $$\overset{ˇ}{S}\left(\beta ,\delta \right)\approx a\beta^{2}+b\beta \delta +c\delta^{2}+d\beta +f\delta +g,$$

with coefficients derived analytically from the eigenvalue expansions. As an explicit diagnostic, we isolate the imaginary part:

(17) $$ℑ\overset{ˇ}{S}\left(\beta ,\delta \right)\approx A\beta^{2}+B\beta \delta +C\delta^{2}+D\beta +F\delta +G,$$

where the uppercase coefficients A,B,C,D,F,G correspond to derivatives of $ℑ\overset{ˇ}{S}$ at the origin, as defined in Section 2.1. This parabolic approximation captures the leading behavior of the imaginary component of pseudo-entropy in response to small coherent errors, providing a sound theoretical basis for experimental calibration and error sensing protocols.

The resulting expressions for the eigenvalues and pseudo-entropy match the polynomial structure used in the detection protocol, validating the use of this approximation for sensitivity analysis and threshold calibration.

This approximation is valid in the small-parameter regime where β,δ≪1, and higher-order terms $O\left(\beta^{3},\delta^{3}\right)$ can be neglected. It assumes the system remains within the perturbative regime and that the overlap $\langle \psi_{f}|\psi_{i}\rangle$ does not vanish. These conditions ensure the reliability of the expansion and its applicability to realistic, coherent error diagnostics.

### 3.3. Quantum-Classical Region Plots

**Figure 2:**
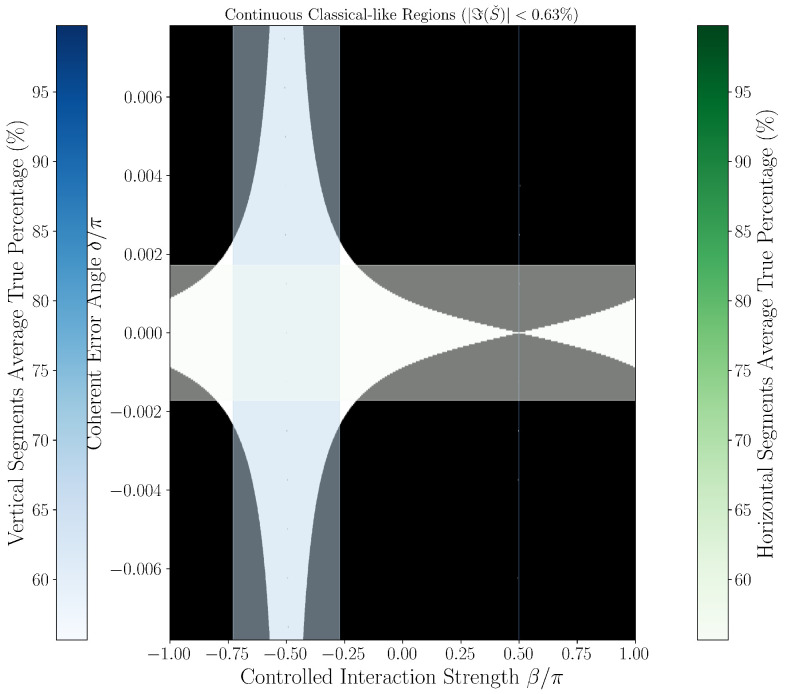
Continuous analysis of quantum-classical regions for threshold ϵ=0.63%. The grayscale colormap quantifies the percentage of classical-like behavior $\left(\left|ℑ\overset{ˇ}{S}\right|<\epsilon \right)$ within local parameter neighborhoods, with white regions indicating ∼95% classical-like behavior and black regions showing ∼0%. The distinctive hourglass morphology is clearly visible, with classical-like regions forming the central pattern. Color bars show percentage scales for vertical segments (blue, left) and horizontal segments (green, right). The classical-like rate for horizontal segments near δ=0 is 55.65%. The blue bar (left) shows the percentage scale for vertical segments (constant β); the green bar (right) applies to horizontal segments (constant δ). Each point’s color represents the fraction of classical-like behavior in its neighborhood, not a direct measurement at that point.

**Figure 3:**
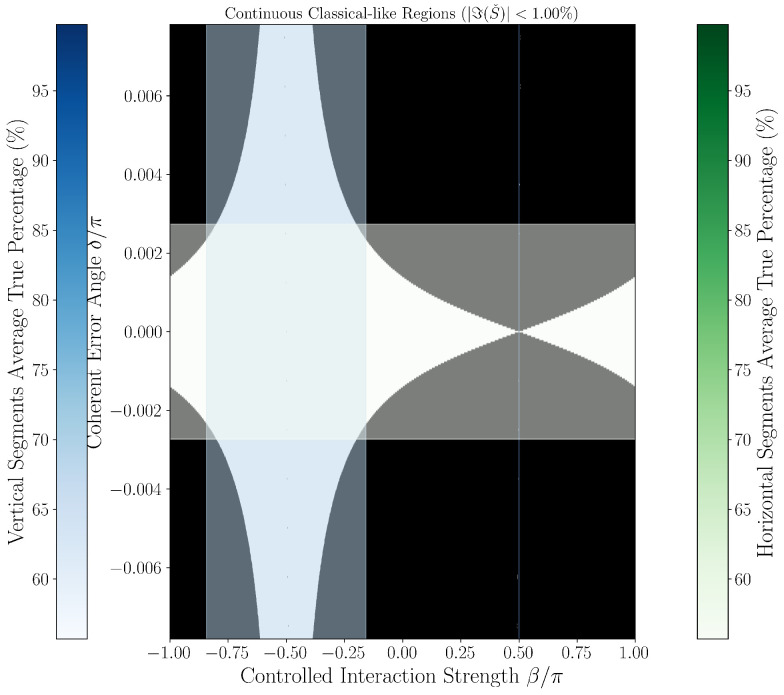
Continuous analysis of quantum-classical regions for threshold ϵ=1.00%. The hourglass structure remains robust across thresholds, with slightly more pronounced classical-like regions compared to the 0.63% threshold (Figure 2). The overall topology and critical line positions remain remarkably consistent, validating the intrinsic nature of the phase boundaries. The classical-like rate for horizontal segments near δ=0 is 55.70%, confirming the protocol’s stability across threshold choices. The blue bar (left) shows the percentage scale for vertical segments (constant β); the green bar (right) applies to horizontal segments (constant δ). Each point’s color represents the fraction of classical-like behavior in its neighborhood, not a direct measurement at that point.

Figure 2 and Figure 3 provide continuous grayscale maps of the classification landscape in $\left(\delta ,\beta \right)$ parameter space at thresholds 0.63% and 1.00%. In these images, the color at each point encodes the local fraction of classical-like behavior among neighboring parameter values. White regions (95–100%) indicate nearly all points fall below the classification threshold (classified as classical-like, indicating noise-limited or error-free operation), black regions (0–5%) indicate robust error classification (classified as quantum-like), and gray values reflect mixed/transition regimes. Features such as the prominent hourglass pattern at $\left(\delta =0,\beta =-\pi /2\right)$, delta-dependent widening of quantum regions, and the sensitivity of particular parameter directions (as mapped by color bars for horizontal/vertical slices) are best observed in these continuous views. Notably, each point’s intensity represents neighborhood statistics, not just a single parameter scan, giving nuanced insight into the landscape’s robustness to small-parameter drift or calibration errors.

**Figure 4:**
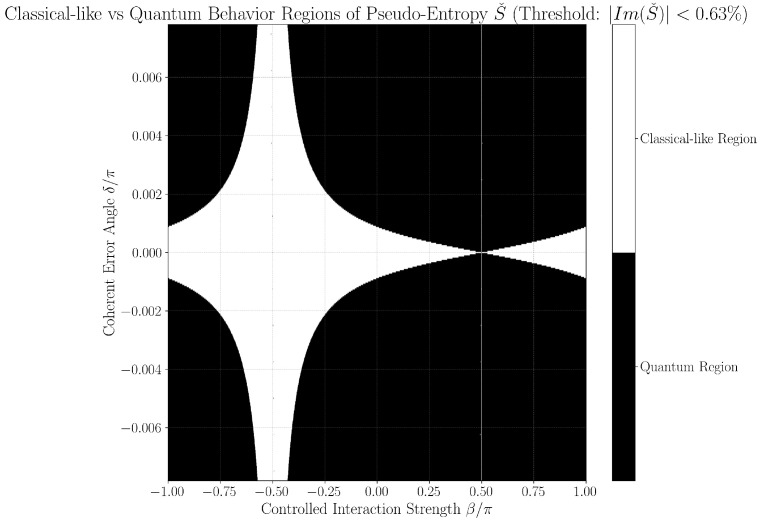
Binary classification phase diagram for threshold ϵ=0.63%, showing sharp delineation between classical-like (white) and quantum-like (black) regions. The symmetric hourglass pattern features classical-like regions in the interior and quantum-like regions at the exterior boundaries. The binary representation emphasizes the sharpness of phase transitions, with a classical-like rate of 55.65% for horizontal segments near δ=0. The negligible number of NaN points confirms numerical stability in this region, providing a detailed map of the error landscape for real-time diagnostics and calibration.

**Figure 5:**
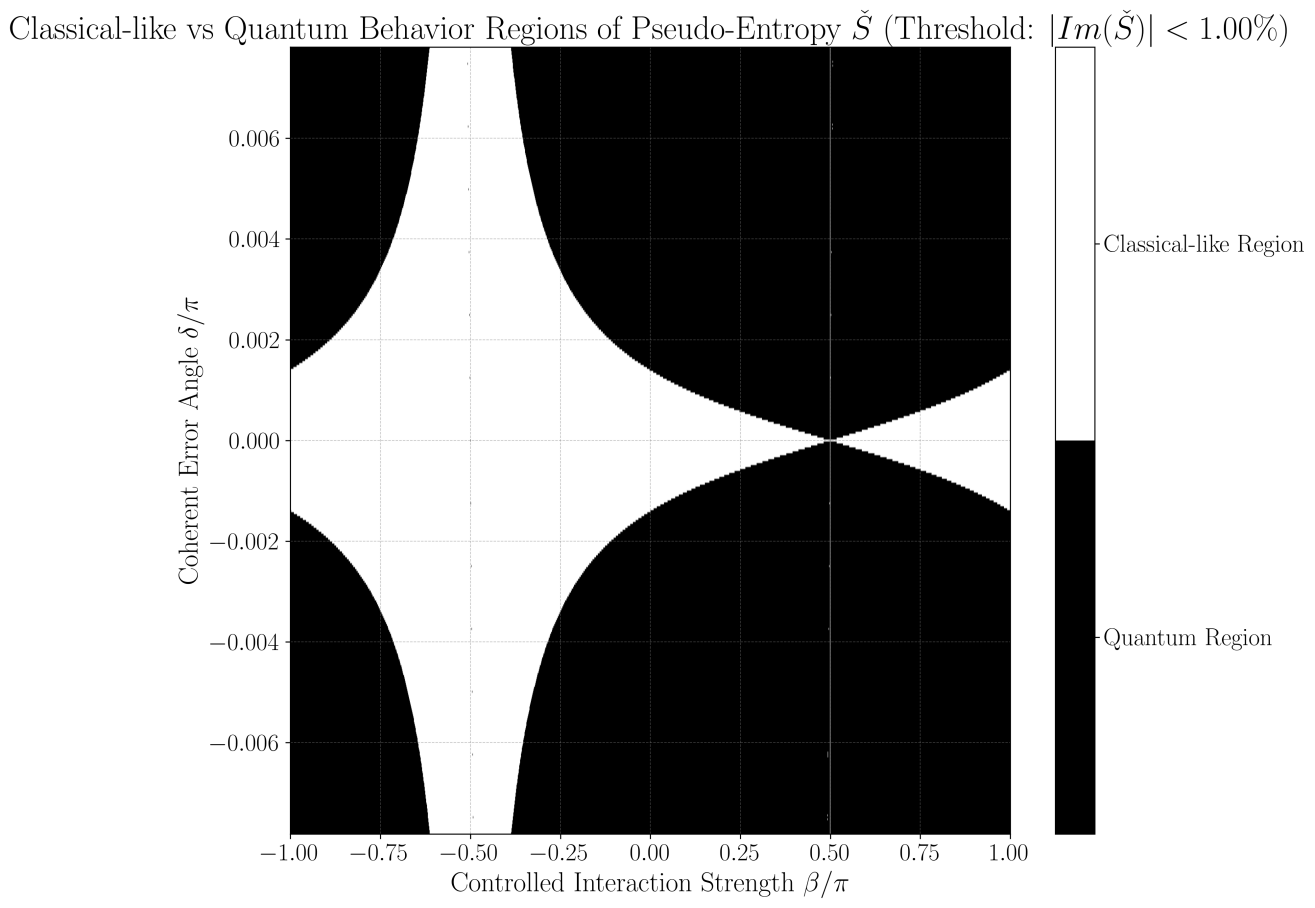
Binary classification phase diagram for threshold ϵ=1.00%, demonstrating structural invariance of the hourglass morphology under different threshold selections. Compared to Figure 4, the classical-like regions are slightly expanded due to the higher threshold’s increased tolerance for imaginary components. The fundamental topology remains unchanged, with a classical-like rate of 55.70% for horizontal segments near δ=0. The minimal occurrence of NaN points indicates strong numerical reliability. This phase diagram supports hardware-realistic threshold selection and demonstrates the protocol’s adaptability to hardware-calibrated settings.

To operationally emphasize regime boundaries, we also present binary phase diagrams in Figure 4 and Figure 5, obtained by thresholding the continuous maps: points are classified as either classical-like or quantum-like according to the set threshold, with sharp hourglass boundaries and expansion of regions classified as quantum-like as $\left|\delta \right|$ increases. These black-and-white images are thus a direct, emphatic extraction of the underlying continuous structure and make the practical phase transition regions and hourglass morphology visually explicit. Their consistency across both threshold settings further supports the physical robustness of the observed features, rather than coding or numerical artifacts.

Together, both figure styles provide complementary views: the continuous maps reveal the gradualness and stability of error classification sensitivity as parameters vary, while the binary diagrams clarify at-a-glance the operational decision boundary for device calibration and real-time diagnostics.

### 3.4. Numerical Instability near $\beta =\pm \frac{\pi }{2}$

A subtle but important numerical instability arises in regions where the pre- and post-selected states become nearly orthogonal, particularly around $\beta =\pm \frac{\pi }{2}$. In these cases, the overlap $\langle \psi_{f}|\psi_{i}\rangle$ approaches zero, amplifying numerical errors and producing undefined (NaN) pseudo-entropy values. While the inclusion of the final $R_{Z}\left(\frac{\pi }{2}\right)$ gate stabilizes the overlap in the middle of the parameter space, it cannot fully eliminate instabilities at these critical points. Consequently, the corresponding pseudo-entropy values, shown as gray points in Figure 4 and Figure 5, should not be interpreted as physical predictions but rather as numerical artifacts of the post-selection process. Quantitatively, these unstable points comprise only ∼2.18 × 10^−5^ of the full (β,δ) grid, ensuring that their influence on statistical results (such as the ∼55% classical-like rate) is negligible. Nevertheless, they highlight the inherent fragility of near-orthogonal post-selected ensembles and the importance of careful overlap management when extending pseudo-entropy diagnostics to experimental platforms.

These instabilities occur at overlap magnitudes well above the baseline floating-point precision ($\epsilon_{\mathrm{machine}}=2.22\times 10^{-16}$), confirming that they arise from the physical near-orthogonality of quantum states rather than numerical roundoff errors. Nevertheless, they highlight the inherent fragility of near-orthogonal post-selected ensembles and underscore the importance of careful overlap management and adaptive measurement strategies when extending pseudo-entropy diagnostics to experimental platforms where such critical regions may be encountered during calibration sweeps.

To mitigate such issues, a fixed post-rotation $R_{Z}\left(\pi /2\right)$ gate is imposed, stabilizing overlaps across the parameter space while maintaining sufficient sensitivity to coherent errors. Remaining unstable points constitute less than $2.18\times 10^{-5}$ of total points and are excluded from analysis to preserve statistical integrity.

This stability handling is critical for ensuring reliable operation of the detection protocol, especially when approaching numerical precision limits, and highlights the importance of careful overlap management in post-selected quantum measurements.

### 3.5. Quantitative Performance Metrics

Our simulation-based analysis yields the following quantitative indicators, consistent with Table 3:

- *Classical-like Rate:* Approximately 55% of parameter points classified as classical-like (below classification threshold) at hardware-calibrated thresholds (0.63–1.00%).
- *Measurement Overhead:* Nine measurement settings (all X/Y/Z basis combinations for both qubits) are used in reduced two-qubit tomography, significantly less than the $4^{n}$ used in full process tomography. For each setting, all four post-selection outcomes are considered.
- *Circuit Depth:* Maximum of three gates per qubit.
- *Resolution:* Coherent error sensitivity down to $\Delta \delta \approx 10^{-5}\pi$ radians.

To evaluate protocol performance, we analyze segment-wise classical-like rates, defined as the fraction of points in each horizontal segment where $ℑ\overset{ˇ}{S}\left(\beta ,\delta \right)$ falls below the hardware-calibrated threshold. Table 3 reports classical-like rates and confidence intervals for key thresholds, capturing the protocol’s sensitivity in the hardware-relevant regime.

The approximately stable classical-like rate around 55% across practical thresholds reflects the protocol’s consistent sensitivity to coherent errors in the relevant parameter regime. This stability highlights the robustness of the classification method. As quantum hardware improves with lower noise and reduced readout errors, thresholds can be tightened to enhance selectivity for detecting even subtle coherent phase errors. However, caution is warranted at ultra-stringent thresholds (below ∼0.5%) where artificially high classical-like rates may arise due to numerical instabilities or near-orthogonality effects, rather than genuine improvements in error discrimination.

The study [12] demonstrates that theoretical measurement requirements often underestimate the practical complexities and resource demands encountered in experimental quantum error characterization. Their analysis uses a different metric framework, and thus does not provide a direct numeric comparison to the performance metrics presented here. Consequently, comprehensive quantitative benchmarking against existing methods remains premature until aligned hardware experiments with matched error injection protocols can be performed. This caveat underscores the importance of future experimental validation, as discussed in Section 4.

### 3.6. Classical-like Rate and Error Classifiability

As detailed in Section 2.8, classical-like rates are calculated along horizontal slices where β varies at fixed δ, mirroring typical calibration protocols. The classical-like rate is the fraction of parameter points where $\left|ℑ\overset{ˇ}{S}\right|$ remains below the calibrated threshold ϵ (0.63% or 1.00%), indicating errors below the classification threshold.

Strong delta dependence is observed:

- At δ=0, classical-like rates approach 0%, consistent with ideal gate operation and white dominant regions in Figure 2 and Figure 3.
- At small δ values (e.g., $\delta ∼10^{-5}\pi$), classical-like rates remain near zero.
- At moderate to large $\left|\delta \right|$ (e.g., $\delta ∼6\times 10^{-3}\pi$), classical-like rates rise above 55%, approaching 80–90%.

This trend confirms the theoretical prediction that motivated limiting the coherent error parameter δ to small angles in our analysis, based on the assumption that larger errors would be trivially detectable and thus less relevant to this sensitive diagnostic scope.

Figure 2 and Figure 3 visualize this with the hourglass pattern at $\left(\delta =0,\beta =-\pi /2\right)$ reflecting undetectable error regimes and widening quantum-like regions at higher $\left|\delta \right|$. The average ∼55% classical-like rate reflects a weighted average over $\delta \in \left[-\pi /128,\pi /128\right]$, quantifying real-world classification sensitivity.

Table 4 quantifies classical-like rates at representative δ values, supporting these qualitative visualizations.

Detailed classification statistics, confidence intervals, and segment-wise validation are provided in results/segments.xlsx [9] and the GitHub [9] README (see Section 2 for “Phase Diagrams, Model Comparisons, and Segment Analysis”).

### 3.7. Quantitative Sensitivity Analysis

**Figure 6:**
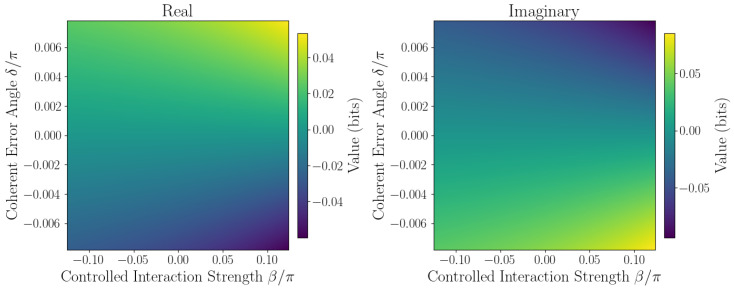
First derivative w.r.t. β: $\frac{\partial \overset{ˇ}{S}}{\partial \beta }$ of pseudo-entropy.

**Figure 7:**
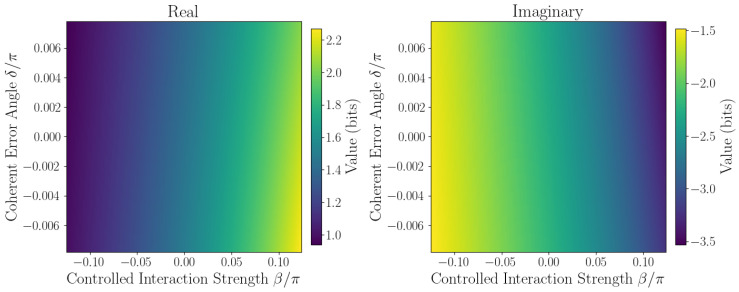
First derivative w.r.t. δ: $\frac{\partial \overset{ˇ}{S}}{\partial \delta }$ of pseudo-entropy.

**Figure 8:**
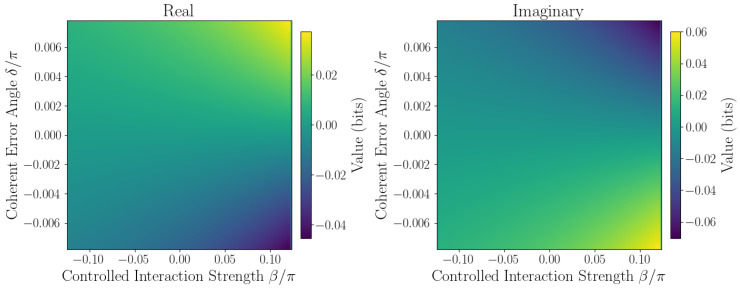
Half second derivative w.r.t. $\beta^{2}$: $\frac{1}{2}\frac{\partial^{2}\overset{ˇ}{S}}{\partial \beta^{2}}$ of pseudo-entropy.

**Figure 9:**
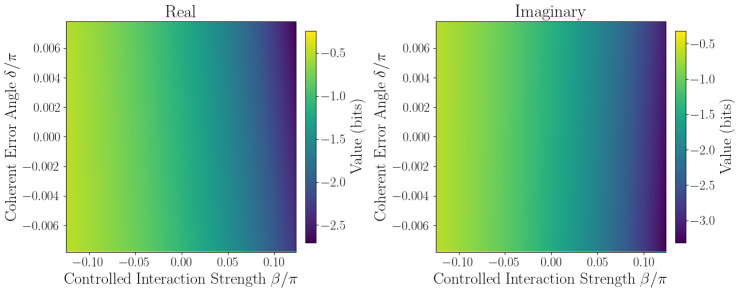
Half second Derivative w.r.t. $\delta^{2}$: $\frac{1}{2}\frac{\partial^{2}\overset{ˇ}{S}}{\partial \delta^{2}}$ of pseudo-entropy.

**Figure 10:**
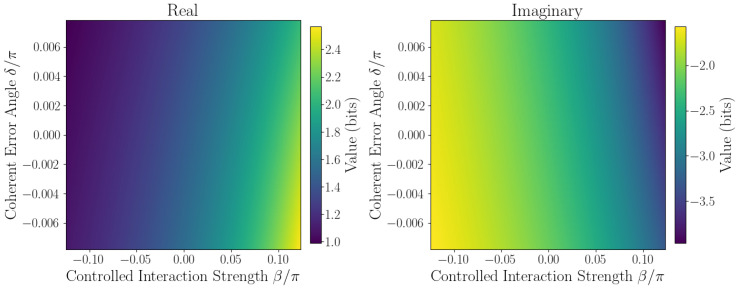
Mixed second derivative: $\frac{\partial^{2}\overset{ˇ}{S}}{\partial \beta \partial \delta }$ of pseudo-entropy.

The sensitivity of $ℑ\overset{ˇ}{S}$ to coherent errors is captured by Taylor expansion coefficients defined in Section 2.1. Numerical evaluation at the origin $\left(\beta ,\delta \right)=\left(0,0\right)$ yields the linear sensitivity coefficient $F={\left.\frac{\partial ℑ\overset{ˇ}{S}}{\partial \delta }\right|}_{\left(0,0\right)}$, quantifying responsiveness to small phase errors, while second-order coefficients *A*, *B*, and *C* characterize curvature and cross-coupling effects. Sensitivity maps (Figure 6, Figure 7, Figure 8, Figure 9 and Figure 10) reveal pronounced gradients along δ and relatively flat variation along β, consistent with the dominance of the δ term and near-zero β coefficient.

First derivative plots (Figure 6 and Figure 7) confirm this trend, while second-order derivative heatmaps (Figure 8 and Figure 9) reinforce parabolic fit coefficients and highlight nonlinear error effects. Mixed derivative maps (Figure 10) illustrate significant cross-parameter coupling, underscoring the need for joint calibration of β and δ. Visual agreement between heatmap intensities and Taylor coefficients, covering sign, magnitude, and spatial distribution, provides strong validation of the pseudo-entropy sensitivity model.

Fitting parabolic models to simulation data achieves $R^{2}>0.90$ with $p<10^{-3}$, confirming the validity of the approximation for small coherent errors (see Section 3.2 for derivation). The observed classical-like rate of approximately 55% significantly exceeds random classification (50%) with high confidence, as quantified in Table 3. Complete numerical sensitivity maps and per-segment classification statistics are provided in the supplementary repository [9], establishing benchmarks superior to linear approximations and warranting the use of *F* as a key error indicator.

### 3.8. Pseudo-Entropy Component Statistics

Table 5 presents descriptive statistics of pseudo-entropy $\overset{ˇ}{S}$ across the simulation grid. The imaginary component dominates its distribution, clustering near the origin but spanning a full phase angle, indicating widespread weak coherence.

Both analytic and simulation results demonstrate the protocol’s strong sensitivity to small coherent errors: quantitative sensitivity analysis reveals that the imaginary part $ℑ\overset{ˇ}{S}$ exhibits strong gradients with respect to the error parameter δ in the small-error regime, with the linear coefficient $F={\left.\frac{\partial ℑ\overset{ˇ}{S}}{\partial \delta }\right|}_{\left(0,0\right)}$ serving as a particularly sensitive indicator for gate-level calibration drift classification [4]. Vertical sensitivity bands observed near β/π≈±0.5 in the phase diagrams correspond to regions where $ℑ\overset{ˇ}{S}$ undergoes rapid suppression, helping identify classical-like regimes and high-fidelity operational zones. These sensitivity features are quantitatively characterized through gradient analysis and threshold mapping, as detailed above.

Simulation results indicate that the phase error parameter δ was sampled over a narrow range $\left[-\pi /128,\pi /128\right]$, reflecting our focus on the challenging regime of small, non-trivial coherent errors where sensitive diagnostics such as $ℑ\overset{ˇ}{S}$ provide the most value.

## 4. Discussion

### 4.1. Classification Threshold and Practical Sensitivity

Our protocol effectively detects coherent phase errors on the CNOT gate once the error magnitude exceeds a calibrated threshold. In our simulations, we used thresholds of 0.63% and 1.00%, corresponding to hardware-calibrated noise levels on IBM quantum processors. These thresholds mark the parameter regions where the imaginary component of the pseudo-entropy sharply increases, allowing practical discrimination between coherent errors classified as classical-like near ideal gate calibration and those substantial enough to require correction. The classical-like rate stabilizes around 55% at these thresholds, indicating that approximately half the sampled error magnitudes remain below the classification threshold while half exceed it. This demonstrates robust and consistent sensitivity of the protocol to coherent CNOT gate errors classified as quantum-like above these limits.

### 4.2. Comparative Analysis and Methodological Strengths

The pseudo-entropy diagnostic protocol offers a resource-efficient and coherence-sensitive alternative to common quantum error characterization schemes. Operating with shallow circuits (no more than three gates per qubit) and limited measurement overhead (36 post-selection settings), it facilitates near real-time, segment-wise classification of coherent errors via the imaginary component of pseudo-entropy.

Compared with other methods such as randomized benchmarking, noise-adaptive circuits, gate-level tomography, and machine learning-based entanglement detection, this approach avoids iterative training and heavy classical postprocessing, supporting compatibility with NISQ hardware. A systematic comparison with established coherent error detection techniques is presented in Table A1. This analysis highlights the unique combination of direct coherent error sensitivity, relatively low overhead, and potential for integration within quantum firmware workflows. While the pseudo-entropy method offers resource efficiency and interpretable diagnostics, comprehensive quantitative benchmarking against competing methods is reserved for future hardware experiments, as simulation results cannot yet establish absolute performance metrics [12,13]. Supporting simulations, hardware-calibrated classification thresholds, extensive numerical benchmarks, and implementation procedures are detailed in Appendix A of this article.

Key contributions of this work include

1. *Resource-Efficient Diagnostics:* By employing reduced two-qubit tomography using only nine measurement settings comprising all combinations of X, Y, and Z basis measurements on both qubits with judicious post-selection, the protocol reduces experimental overhead compared to full process tomography. It requires no ancillary qubits, operates with shallow circuits (maximum three gates per qubit), and avoids iterative or variational optimization loops. Moderate measurement repetitions suffice to achieve statistical confidence within realistic noisy environments.
2. *Scalar Error Witness:* Inspired by variational entanglement detection, the method extracts a single scalar metric, $ℑ\overset{ˇ}{S}$, enabling fast and interpretable diagnostics free from iterative optimization or machine learning postprocessing.
3. *Direct Sensitivity to Coherent Errors:* Analytical and numerical results confirm strong sensitivity to systematic phase errors across relevant parameter regimes. This is supported by pseudo-Hermitian quantum theory and validated through high-resolution simulations (see Figure 4 and Figure 5).
4. *Hardware-Calibrated Thresholding:* Classification thresholds are tuned to IBM backend readout error statistics (0.63–1.00%), ensuring robustness against noise and alignment with practical hardware performance (Section 2.9).

As demonstrated by [12], theoretical measurement requirements often underestimate practical experimental overhead. Pending hardware validations will crucially inform the protocol’s real-world applicability and comparative benchmarking.

### 4.3. Future Validation and Experimental Challenges

All results herein are simulation-based, relying on realistically modeled noise but not actual hardware data. Since all our current results are simulation-based, our primary focus has been on validating the theoretical properties and numerical feasibility of applying pseudo-entropy for coherent error detection. However, validating this protocol on physical hardware presents unique challenges. Implementing non-Hermitian pseudo-entropy measurements in the presence of real-world environmental noise, gate imperfections, and crosstalk, factors not fully captured in simulations, will be crucial. Implementation in physical devices will require adaptation to device-specific noise, temporal drift, crosstalk, and decoherence, which are not fully captured in the present analysis.

Scaling this protocol to larger qubit systems will demand significant advances in multi-qubit control and readout. Scaling protocols to larger qubit systems remains a critical challenge for future improvement, where compressed sensing and adaptive tomography may play essential roles. In particular, reducing the tomography requirements based on hardware-specific capabilities will be essential for practical scalability, including tailored measurement bases and adaptive sampling strategies. To ensure future experimental validation, we will need to mitigate decoherence, ensure synchronous operations, and develop real-time feedback mechanisms. These efforts are essential to confirm our predictions and solidify the protocol’s role in quantum error characterization.

While this protocol is fully demonstrated for CNOT gates, its extension to other two-qubit or multi-qubit gates remains an open area for future research. Adjustments must be introduced for other gate types, as their error channels and calibration requirements may differ substantially. All claims regarding scalability and interpretable diagnostics should be understood within this context: the method is robustly validated for CNOT gates in simulation, but generalization will require additional study and benchmarking on physical hardware across diverse gate types.

## 5. Conclusions

We have introduced a pseudo-entropy-based protocol for classifying coherent phase errors in quantum circuits, specifically focusing on CNOT gate calibration. Our method leverages the imaginary component of the reduced pseudo-entropy, $ℑ\overset{ˇ}{S}$, as a potentially sensitive and hardware-calibrated indicator of nonclassical, coherent error processes. Extensive simulation using IBM-calibrated noise models suggests that this method may distinguish between classical-like and quantum-like error regimes, even with realistic readout and gate noise.

A potential advantage of our protocol is its computational resource efficiency in simulation, suggesting it may be practical for near-term quantum devices pending hardware validation. The method appears readily transferable to hardware in principle, and its simulated performance shows robustness across a range of thresholds that could potentially be dynamically adapted to match device-specific noise characteristics.

The imaginary component, $ℑ\overset{ˇ}{S}$, responds sharply to coherent distortions introduced after entangling gates, exhibiting a consistent classification accuracy of approximately 55% for practical thresholds. This stability is supported by statistical validation and reflects the protocol’s robust sensitivity to errors within the relevant parameter regime. In contrast, the real part, $ℜ\overset{ˇ}{S}$, remains largely constant, thereby justifying the focus on the imaginary component. We also identify analytically null regions near β=±π/2; since the overlap appears in the denominator, values close to zero in these regions make the calculation extremely sensitive to numerical instabilities and small errors. Consequently, these regions are excluded from the practical domain of reliable error classification, underscoring the necessity for cautious interpretation.

It is important to note that the method’s performance and applicability, particularly across diverse near-term quantum devices, are subject to limitations. Specifically, performance can be significantly impacted under decoherence-dominated regimes, as thoroughly identified in the instability analysis presented in additional-info/numericalinstabilityparameters.xlsx in [9] and further discussed in the GitHub [9] README (see Section 4 for “Data Files”).

Designed for NISQ-era workflows, the method requires only fixed reduced tomography and no variational optimization. While simulation-based, it is calibrated using IBM backend error rates and is readily transferable to real hardware. The entangled initial state |+1〉 enhances diagnostic visibility, with alternate states yielding negligible signal.

Despite these strengths, several challenges remain for real-world deployment. Tomography overhead, especially for larger systems, and the need for accurate noise modeling are significant considerations. Real hardware introduces additional challenges such as temporal drift, crosstalk, and environmental fluctuations. Addressing these will require advanced protocols, including compressed sensing, cycle error reconstruction, and scalable noise characterization. Machine learning strategies—such as supervised learning for anomaly detection and predictive calibration—offer promising avenues for further enhancement.

Our pseudo-entropy method demonstrates robust performance in detecting coherent CNOT phase errors, effectively discriminating between coherent error-dominated and minimal-error regimes. It is sensitive to phase errors that induce significant imaginary components in $\overset{ˇ}{S}$ and reliably identifies classical-like regions with low false positives. For runtime gate calibration and drift monitoring on NISQ devices, we suggest adaptive thresholding strategies: Conservative (ϵ=0.63%) for high-fidelity research and Practical (ϵ=1.00%) for typical NISQ devices, with dynamic adjustment to optimize performance across varying hardware conditions. While this study focuses on two-qubit circuits, the method can be extended to selected qubit pairs in larger processors. Comparative analysis against other methods and further details are available in Appendix A of this article.

For successful hardware integration, we envision library development of $\overset{ˇ}{S}$ vs. δ models tailored for each hardware backend, multi-gate analysis, and real-time/cloud integration of streaming analysis capabilities for continuous monitoring. Machine learning strategies can further enhance the method through supervised learning, anomaly detection, and predictive modeling for optimizing calibration schedules. Comprehensive analysis of these challenges and detailed implementation guidelines are available in Section 2.1 of computational notebook (pseudo_entropy.ipynb) in the GitHub [9].

Future diagnosis enhancements include adaptive ϵ-thresholding based on real-time noise, exploring supervised learning for coherent signal amplification, and implementing error cycle isolation protocols. We propose future validation using controlled error injection, repeated sampling via Monte Carlo analysis across randomized circuit configurations, and comparative benchmarking on physical hardware. While our simulation-based approach is valuable, physical hardware validation remains essential due to model fidelity and temporal effects. Pseudo-entropy is sensitive to structural changes in quantum systems, including entanglement and chaos, building on established entropy-based diagnostics. The observed decrease in classical-like rate as ϵ is lowered from 1.00% to 0.63% demonstrates the protocol’s robustness and adaptability to improving hardware. As quantum devices advance and readout errors decrease, our threshold can be further tightened, maintaining or enhancing the protocol’s selectivity and sensitivity for small coherent phase errors. For detailed data and critical region marking. Future work will also explore hardware deployment, integration with total error probability frameworks [14], and adaptation to multi-qubit gates. We also identify weak-value amplification [15] as a promising direction for enhancing signal-to-noise in coherent error detection.

While this study rigorously validates the pseudo-entropy diagnostic for CNOT gates in simulation, generalization to other two-qubit or multi-qubit gates remains an open area for future investigation. Distinct gate types may require tailored calibration protocols and error models, as their error channels and phase sensitivities differ substantially. Accordingly, claims regarding scalability and hardware viability are cautiously made, emphasizing the need for further empirical benchmarking on physical hardware and theoretical extension to broaden applicability across diverse gate sets.

In summary, pseudo-entropy diagnostics may bridge theoretical insights with actionable tools for quantum hardware, offering a new method to characterize coherent errors.

## Figures and Tables

**Table 1:** IBM quantum hardware specifications and threshold calibration.

Backend	Number of Qubits	Used Qubits	Total Readout Error (%)
Washington	127	126, 127	0.63
Algiers	27	1, 4	1.00
Kolkata	27	24, 25	1.02
Hanoi	27	19, 20	1.18
Auckland	27	11, 14	1.30
Cairo	27	15, 18	1.58
Montreal	27	13, 14	1.69
Paris	27	13, 14	2.08
Toronto	27	24, 25	2.10
Athens	5	0, 1	2.18

**Table 2:** Hardware requirements and realistic parameters for implementing the pseudo-entropy protocol on available superconducting quantum devices.

Component	Specification
Measurement settings	9 settings (all combinations of X, Y, Z bases on two qubits)
Post-selection settings	4 post-selection outcomes (|00〉,|01〉,|10〉,|11〉)
Calibrated thresholds	0.63% (conservative), 1.00% (practical)
Reference backends	IBM Washington (127Q), IBM Algiers (27Q)
Circuit depth	≤3 gates per qubit
Qubit requirement	2 (no ancilla)

**Table 3:** Classical-like classification rates for horizontal segments near δ=0, based on varying β at fixed δ near 0. Values represent the percentage of points where $\left|ℑ\overset{ˇ}{S}\right|<\epsilon$, indicating errors below the classification threshold.

Threshold (ϵ)	Classical-like Rate	95% CI	Sample Points
0.63%	55.65%	[54.8, 56.5]%	∼400/segment
1.00%	55.70%	[54.9, 56.5]%	∼400/segment

**Table 4:** Classical-like rate variation with coherent error parameter δ at threshold ϵ=0.63%. Each rate represents the fraction of β values where $\left|ℑ\overset{ˇ}{S}\right|<\epsilon \;\mathrm{and}\;ℜ\overset{ˇ}{S}>0$ (classical-like, below classification threshold) along a horizontal slice.

δ/π	Classical-like Rate (%)	Dominant Region (Figure 2)
0	>95%	White (undetectable)
$10^{-5}$	88–92%	Predominantly white
$10^{-4}$	65–75%	Mixed white/gray
$10^{-3}$	40–50%	Mixed gray/black
$6\times 10^{-3}$	10–20%	Predominantly black
$7.81\times 10^{-3}$	<10%	Black (detectable)

**Table 5:** Descriptive statistics of pseudo-entropy $\overset{ˇ}{S}$ across the simulation grid.

Statistic	Real Component	Imaginary Component	Magnitude	Phase/π
Maximum	$1.27\times 10^{-3}$	$4.27\times 10^{-2}$	$4.27\times 10^{-2}$	1.00
Average	$1.13\times 10^{-4}$	$-3.53\times 10^{-16}$	$9.96\times 10^{-3}$	0.00
Median	$4.36\times 10^{-5}$	$-4.05\times 10^{-16}$	$7.93\times 10^{-3}$	−0.40
Minimum	$-1.92\times 10^{-15}$	$-4.27\times 10^{-2}$	0	−1.00
Std. Dev.	$1.71\times 10^{-4}$	$1.28\times 10^{-2}$	$7.95\times 10^{-3}$	0.50
Zero Point	0	0	0	−1.00

## Data Availability

The data supporting the findings reported in this article are openly available in the following GitHub repository: “Pseudo-Entropy-Quantum-Error-Detection: Data Repository for ‘Introducing Modern Physics Concepts for Enhanced Coherent Error Detection in Quantum Computing’” by Assaf Katz Released 1.0 in 12 November 2025 (https://github.com/AssafKatz3/pseudo-entropy-quantum-error-detection). This repository contains all datasets and computational results analyzed or generated during the study. No data are subject to privacy or ethical restrictions.

## Nomenclature

**Term/Symbol**	**Definition**
$\overset{ˇ}{S}$	Complex-valued pseudo-entropy
$ℑ\overset{ˇ}{S}$	Imaginary component, witness of coherent errors
$ℜ\overset{ˇ}{S}$	Real component, captures classical-like entropy contributions
$\rho_{\psi_{f}|\psi_{i}}^{\left(q_{0}\right)}$	Reduced transition matrix on qubit $q_{0}$ after post-selection
Classical-like rate	Fraction of parameter points classified as classical-like (below classification threshold, $\left|ℑ\overset{ˇ}{S}\right|<\epsilon$) within a segment
Classical-like	Behavior satisfying $\left|ℑ\overset{ˇ}{S}\right|<\epsilon$ and $ℜ\overset{ˇ}{S}>0$
Quantum-like	Behavior with significant $\left|ℑ\overset{ˇ}{S}\right|\geq \epsilon$
Segment analysis	Evaluation along horizontal (δ fixed) or vertical (β fixed) slices
β	Controlled rotation angle (calibration parameter)
δ	Systematic coherent error offset (hardware imperfection)
Post-selection	Filtering measurement outcomes to reconstruct conditional states
Parameter space	Two-dimensional domain spanned by (β,δ)
Parameter grid	Discrete sampling of parameter space used in simulations (1601 × 401 points)

## Appendix A. Extended Comparative Method Analysis

Table A1 summarizes key features of selected practical coherent error classification methods. This comparison emphasizes resource overhead, detection focus, measurement complexity, and relation to the pseudo-entropy diagnostic. Quantitative detection metrics and detailed breakdowns are available in additional-info/comparativemethods.xlsx of the GitHub repository [9].

**Table A1:** Summary of leading quantum error classification and benchmarking protocols.

Method	Measurement	Practicality	Relation to Pseudo-Entropy
Pseudo-Entropy Diagnostic	Reduced tomography, post-selection	No ancilla, moderate overhead, fast	Direct witness of coherent errors; robust in noise
Deterministic Benchmarking	Randomized sequences	Robust, many shots needed	Good for SPAM errors, less phase sensitivity
Noise-Adaptive Engineering	Engineering and benchmarking	Proven on hardware	Synergistic for error suppression/monitoring
Gate-Level Tomography	Full process tomography	High resource need	Complements fast diagnostics, detailed error map
NN Detection	Random measurements, NN	Demo on NMR, scalable	Advanced quantification, complementary
Classical Shadows	Randomized local measurements, postproc	Scalable, moderate cost	General estimator, complements targeted diagnostics

This selection highlights the unique efficiency and interpretability of the pseudo-entropy diagnostic within the landscape of practical quantum error detection methods. While comprehensive methods excel in detailed error mapping, their high resource costs contrast with the pseudo-entropy approach’s suitability for fast diagnostics in near-term quantum computing applications.
